# Supplementary material for: The Association Between Exposure to COVID-19 and Mental Health Outcomes Among Healthcare Workers
Source: Front Public Health. 2022 Jun 10;10:896843. doi: 10.3389/fpubh.2022.896843 (PMC9226479; doi:10.3389/fpubh.2022.896843)
Supplement: Supplementary file 1 [file Table_1.DOCX]

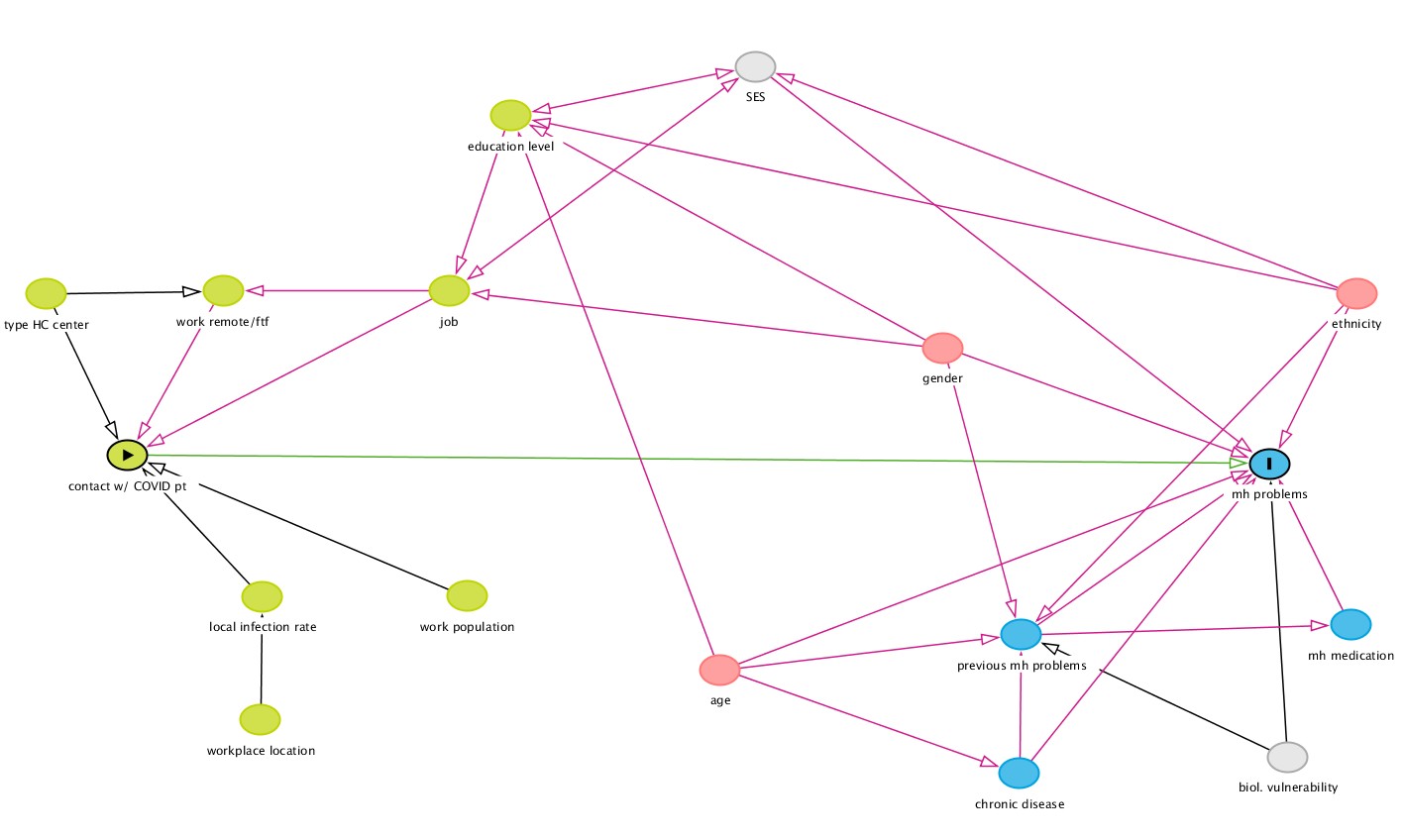


**Supplementary figure 1** | DAG with having contact with COVID-19 patient(s) as the exposure and mental health problems as the outcome


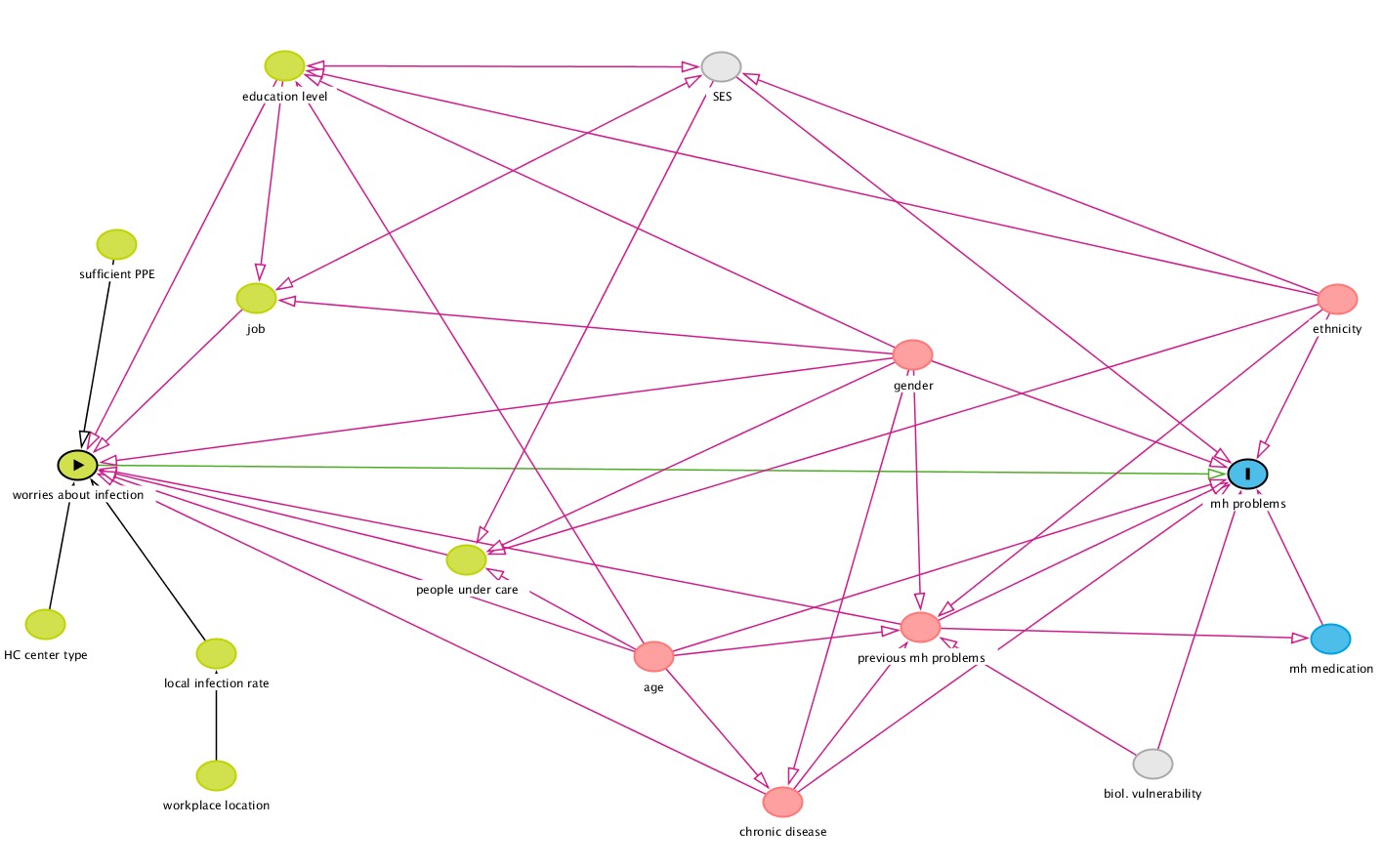


**Supplementary figure 2** | DAG with worries about infections as the exposure and mental health problems as the outcome


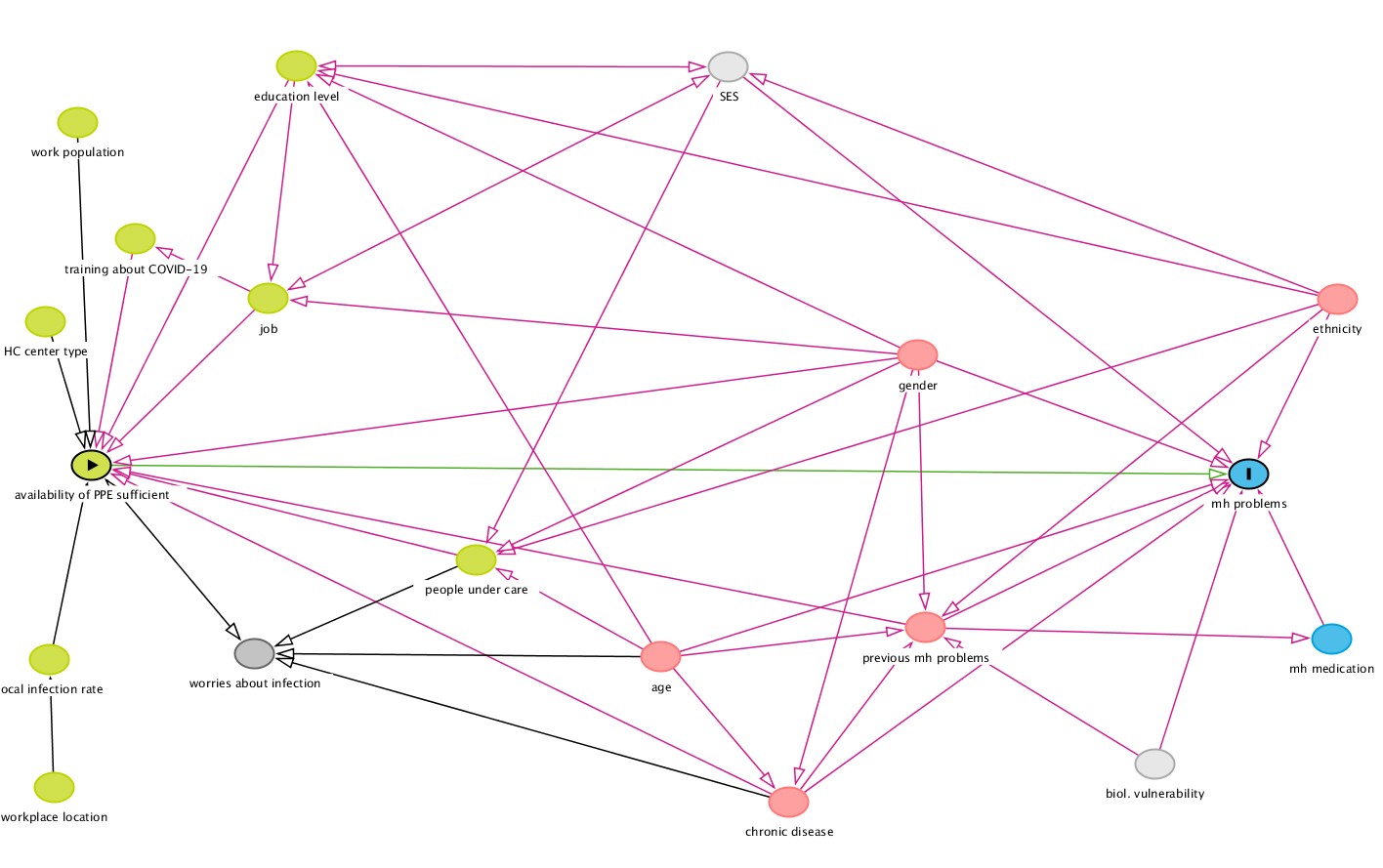


**Supplementary figure 3** | DAG with availability of sufficient PPE as the exposure and mental health problems as the outcome


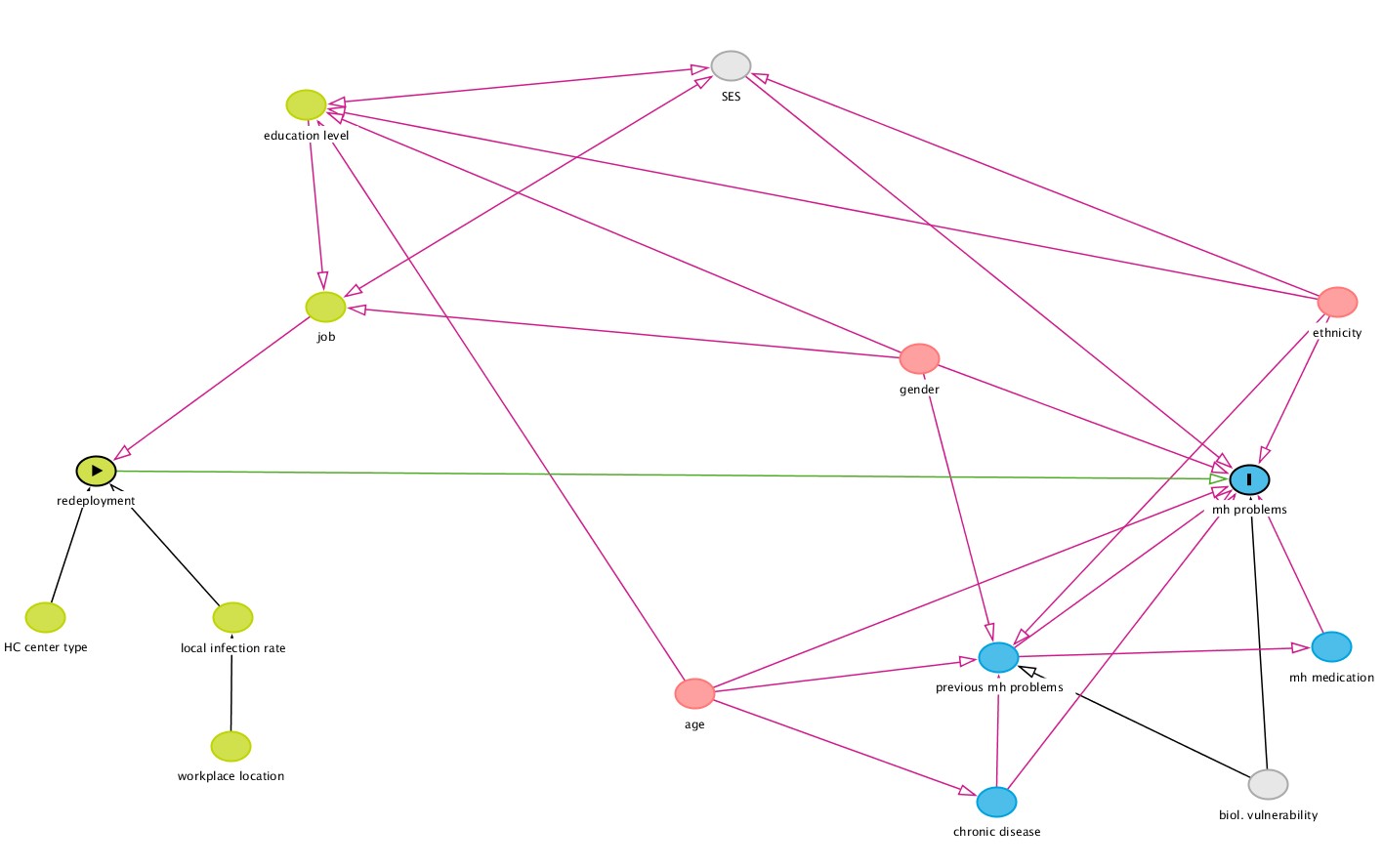


**Supplementary figure 4** | DAG with redeployment as the exposure and mental health problems as the outcome

| **Supplementary Table 1** \| Distribution of COVID-19 infection and death rates per province around the time of participant recruitment | | | | | | | | | | |
| --- | --- | --- | --- | --- | --- | --- | --- | --- | --- | --- |
| Province |  | Time period | | | | | | | | |
|  |  | January 2021 | |  | March 2021 | |  | May 2021 | |  |
|  |  | Infection rates | Death  rates |  | Infection rates | Death  rates |  | Infection rates | Death rates |  |
| Drenthe |  | 1.013 | 21,22 |  | 998 | 7,07 |  | 205 | 2,22 |  |
| Flevoland |  | 995 | 8,87 |  | 778 | 3,27 |  | 232 | 2,34 |  |
| Friesland |  | 1.020 | 13,82 |  | 984 | 8,14 |  | 157 | 1,84 |  |
| Gelderland |  | 1.116 | 16,07 |  | 908 | 4,01 |  | 53 | 2,29 |  |
| Groningen |  | 991 | 9,88 |  | 778 | 4,77 |  | 169 | 1,87 |  |
| Limburg |  | 1.429 | 24,20 |  | 1.003 | 7,62 |  | 128 | 4,84 |  |
| N. Brabant |  | 1.080 | 10,72 |  | 1.130 | 4,78 |  | 42 | 2,14 |  |
| N. Holland |  | 986 | 13,26 |  | 1.184 | 5,44 |  | 34 | 2,84 |  |
| Overijssel |  | 1.336 | 22,38 |  | 828 | 3,17 |  | 115 | 1,80 |  |
| Utrecht |  | 874 | 10,95 |  | 957 | 4,33 |  | 64 | 2,50 |  |
| Zeeland |  | 1.013 | 8,04 |  | 1.103 | 11,42 |  | 263 | 3,37 |  |
| S. Holland |  | 880 | 15,24 |  | 1.187 | 6,87 |  | 24 | 3,33 |  |
| *Note.* N. Brabant = North Brabant, N. Holland = North Holland, S. Holland = South Holland. The reported infection and death rates are the number of confirmed COVID-19 cases and COVID-19 related deaths per 100.000 inhabitants in the following time periods: January 1^st^ 2021 - January 31^st^ 2021, March 1^st^ 2021 -March 31^st^ 2021 and May 1^st^ 2021 - May 31^st^ 2021 respectively. | | | | | | | | | | |

| **Supplementary Table 2** \| Grouping of specific professions in job categories | |
| --- | --- |
| Job category | Specific professions |
| Physicians | Physicians, including residents and medical specialists |
| Nursing staff | Nurses, including nursing assistants and nursing technicians |
| Other clinical specialists & managers | Psychologists, social workers, physical therapists, respiratory therapists, dietitians, dentists, occupational therapists, midwives, speech therapists, paramedics, pharmacists, biologists, and clinical managers |
| Support & ancillary staff | Radiology technicians, laboratory technicians, secretary, patient transportation, cleaning staff, food/hospitality, maintenance staff and security staff |
| Other HCW’s | Coaches, buddies, spiritual counselors, researchers, trainers and advisors, non-clinical managers |

| **Supplementary table 3** \| Parsimonious multilevel models for psychological distress, depressive symptoms and PTSS | | | | | | | | | |
| --- | --- | --- | --- | --- | --- | --- | --- | --- | --- |
|  | | Psychological distress | |  | Depressive symptoms | |  | PTSS | |
| Variables |  | β (95% CI) | SE |  | β (95% CI) | SE |  | β (95% CI) | SE |
| Intercept | | 12.15** (6.44-17.85) | 2.80 |  | 4.22* (-.70-7.75) | 1.75 |  | 1.56* (.44-2.67) | .55 |
| Individual level | |  |  |  |  |  |  |  |  |
| Female | | .42 (-.67-1.51) | .55 |  | .49 (-.28-1.26) | .39 |  | .19 (-.05-.43) | .12 |
| Other gender | | .43 (-6.15-7.01) | 3.31 |  | -69 (-5.23-3.86) | 2.29 |  | .82 (-.97-2.61) | .89 |
| Postgraduate   studies | | -.99 (-6.55-4.58) | 2.73 |  | .13 (-3.36-3.61) | 1.72 |  | -.47 (-1.48-.55) | .50 |
| Undergraduate   degree | | -1.28 (-6.64-4.07) | 2.63 |  | .04 (-3.31-3.39) | 1.66 |  | -.21 (-1.25-.83) | .51 |
| Techn.- profess.   training | | -1.54 (-7.02-3.93) | 2.68 |  | .05 (-3.49-3.59) | 1.75 |  | -.33 (-1.34-.68) | .50 |
| Secondary school | | .64 (-4.67-5.96) | 2.63 |  | 1.69 (-2.02-5.41) | 1.84 |  | -.30 (-1.43-.84) | .56 |
| Physicians | | .88 (-1.25-3.02) | 1.08 |  | -1.15 (-2.68-.39) | .77 |  | -.33 (-.81-.14) | .24 |
| Nursing staff | | 1.47 (-.17-3.11) | .83 |  | -.50 (-1.85-.85) | .68 |  | -.10 (-.49-.29) | .20 |
| Other clinical   spec. & managers | | 1.23 (-.45-2.90) | .85 |  | -.29 (-1.73-.1.15) | .73 |  | -.22 (-.60-.17) | .19 |
| Support &   auxiliary staff | | .63 (-1.17-2.43) | .91 |  | -1.08 (-2.54-.38) | .74 |  | -.26 (-.68-.16) | .21 |
| Having someone  under care | | -.19 (-1.02-.63) | .42 |  | .02 (-.60-.63) | .31 |  | .07 (-.11-.26) | .09 |
| Chronic physical   illness | | 1.65* (.62-2.69) | .53 |  | .80 (-.01-1.60) | .41 |  | .18 (-.05-.41) | .12 |
| Previous mental  health problems | | .78 (-1.04-2.59) | .90 |  | 1.24 (-.46-2.94) | .83 |  | -.04 (-.47-.39) | .21 |
| Age | | -.02 (-.05-.02) | .02 |  | -.02 (-.04-.01) | .01 |  | -.00 (-.01-.01) | .00 |
| Contact with   COVID patient(s) | | -.08 (-1.11-.95) | .52 |  | -.09 (-.86-.69) | .39 |  | .14 (-.08-.35) | .11 |
| Being worried   about infection | | .81** (.52-1.11) | .15 |  | .79 ** (.57-1.00) | .11 |  | .32** (.26-.39) | .03 |
| Sufficient PPE | | -.78* (-1.36-.19) | .29 |  | -.45 (-.91-.00) | .23 |  | -.10 (-.22-.02) | .06 |
| Redeployed | | -.01 (-1.17-1.14) | .58 |  | .60 (-.27-1.46) | .44 |  | .07 (-.20-.34) | .14 |
| *Note.* Techn.- profess. training = technical- professional training, other clinical spec. & managers= other clinical specialists and managers, having someone under care = having a minor, older adult, or individual with a disability under care.  * *p* < .05, ** *p* < .001. | | | | | | | | | |
